# P-127. Safety and Efficacy of Fecal Microbiota Spores, Live-brpk (Formerly SER-109) in Patients with Recurrent *Clostridioides difficile* Infection (CDI): Subgroup Analysis by Number of Prior CDI Recurrences from an Integrated Analysis of Phase 3 Trials

**DOI:** 10.1093/ofid/ofae631.332

**Published:** 2025-01-29

**Authors:** Matthew Sims, Colleen S Kraft, Edward Huang, Lisa von Moltke, Brooke Hasson, Maximilian von Eynatten, Darrell Pardi

**Affiliations:** Corewell Health William Beaumont University Hospital, Oakland University William Beaumont School of Medicine, Royal Oak, Michigan; Emory University, Atlanta, GA; Sutter Health Research Enterprise, San Jose, California; Seres Therapeutics, Cambridge, Massachusetts; Seres Therapeutics, Cambridge, MA, Cambridge, Massachusetts; Nestlé Health Science, Vevey, Vaud, Switzerland; Mayo Clinic, Rochester, MN

## Abstract

**Background:**

Patients experiencing ≥1 recurrence of CDI are at elevated risk for subsequent recurrences and complications versus those with an initial CDI episode. Treatment selection for recurrent CDI (rCDI) is influenced by the number of recurrences a patient has experienced. Fecal microbiota spores, live-brpk (VOWST™; formerly SER-109, hereafter referred to as VOS for Vowst Oral Spores) is an oral microbiota-based therapeutic to prevent rCDI in adults with rCDI after antibacterial treatment for rCDI, thought to work by enabling rapid restoration of the microbiome. We report results from an integrated analysis of two Phase 3 trials to further describe safety/efficacy of VOS in patients, stratified by number of prior CDI recurrences.

**Figure:**
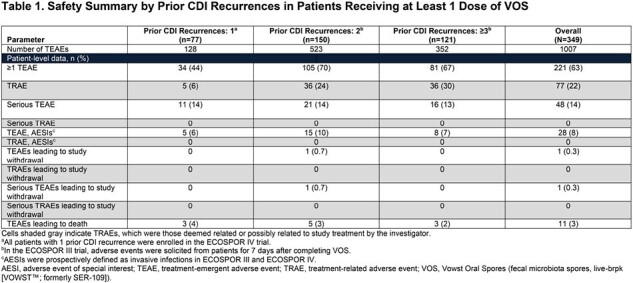

**Methods:**

The randomized, controlled ECOSPOR III trial enrolled 182 patients with ≥2 CDI recurrences, and the open-label, single-arm ECOSPOR IV trial enrolled 263 patients with ≥1 CDI recurrence. VOS was given as 4 capsules over 3 consecutive days after completing antibiotic treatment. Treatment-emergent adverse events (TEAEs) were collected through Week 8 after VOS; serious TEAEs/AEs of special interest were collected through Week 24. Efficacy endpoint included rCDI (toxin-positive diarrhea requiring treatment) through Week 8.

**Results:**

A total of 77 patients had 1, 150 patients had 2, and 121 patients had ≥3 prior CDI recurrences; incidence of TEAEs was 44%, 70%, and 67%, respectively (**Table 1**). No serious TEAEs, TEAEs of special interest, or TEAEs leading to study withdrawal were considered related to VOS by investigators. TEAEs in ≥15% of patients with 1, 2, or ≥3 prior CDI recurrences were flatulence (6%; 29%; 29%), abdominal pain (6%; 27%; 16%), fatigue (1%; 26%; 20%), abdominal distension (3%; 22%; 20%), and diarrhea (27%; 21%; 23%). On-study recurrence rate through Week 8 in the overall population was 9.5% (95% CI: 6.6%–13.0%); in those with 1 or multiple (≥2) CDI recurrences, rates (95% CI) were 6.5% (2.1%–14.5%) and 10.3% (7.0%–14.6%), respectively.

**Conclusion:**

This integrated analysis confirms VOS was well tolerated and rCDI rates were low/consistent in patients, regardless of the number of CDI recurrences. Results suggest VOS may be considered in patients with rCDI, regardless of the number of prior CDI recurrences.

**Disclosures:**

**Matthew Sims, MD, PhD**, Adaptive Phage Therapeutics: Grant/Research Support|Applied Biocode: Advisor/Consultant|Applied Biocode: Grant/Research Support|Astra Zeneca: Grant/Research Support|Biotest AG: Grant/Research Support|ContraFect: Grant/Research Support|Janssen: Grant/Research Support|Leonard-Meron Biosciences: Grant/Research Support|Novozyme: Grant/Research Support|OpGen: Advisor/Consultant|OpGen: Grant/Research Support|Pfizer: Grant/Research Support|Prenosis: Advisor/Consultant|Prenosis: Grant/Research Support|QIAGEN: Grant/Research Support|Seres: Advisor/Consultant|Seres: Grant/Research Support|Summit Therapeutics: Grant/Research Support|Venatorx: Advisor/Consultant **Colleen S. Kraft, MD, MSc**, Ferring: Advisor/Consultant|Seres: Advisor/Consultant **Lisa von Moltke, MD**, Cara Therapeutics: Board Member|Cara Therapeutics: Stocks/Bonds (Public Company)|Seres Therapeutics: Employee|Seres Therapeutics: Stocks/Bonds (Public Company) **Brooke Hasson, PhD**, Seres Therapeutics: employee|Seres Therapeutics: Stocks/Bonds (Public Company) **Maximilian von Eynatten, MD**, Nestlé Health Science: Employment **Darrell Pardi, MD**, AMT, Inc: Grant/Research Support|Boehringer Ingelheim: Grant/Research Support|Exe GI: Grant/Research Support|Immunic: Advisor/Consultant|Lilly: Advisor/Consultant|Rise Therapeutics: Advisor/Consultant|Rise Therapeutics: Grant/Research Support|Seres Therapeutics: Advisor/Consultant|Seres Therapeutics: Expert Testimony|Vedanta: Advisor/Consultant|Vedanta: Grant/Research Support

